# Effect of exercise on myocardial energy metabolism and relationship between coronary microvascular dysfunction and abnormal myocardial energetics in diabetic cardiomyopathy

**DOI:** 10.1186/1532-429X-17-S1-O98

**Published:** 2015-02-03

**Authors:** Eylem Levelt, Chris Rodgers, William T Clarke, Masliza Mahmod, Rina Ariga, Jane M Francis, Alexander Liu, Cameron Holloway, Matthew D Robson, Kieran Clarke, Theodoros D Karamitsos, Stefan Neubauer

**Affiliations:** 1 OCMR, University of Oxford, Oxford, UK; 2 Department of Physiology, Anatomy&Genetics, University of Oxford, Oxford, UK

## Background

Diabetic cardiomyopathy is a well-recognised entity, with multifactorial aetiology. We and others have previously shown impaired myocardial energetics (decreased PCr/ATP using cardiac 31P Magnetic Resonance Spectroscopy-MRS) in patients with type II diabetes mellitus (T2DM) at rest. Furthermore, diabetic patients are known to have impaired myocardial perfusion reserve even in the absence of epicardial coronary artery disease likely due to microvascular dysfunction. However, it is unknown whether this hypoperfusion is translated to further energetic derangement at stress.

## Methods

27 patients (13 male, mean age 54±1.5 years; BMI 28±2.7) with T2DM and 24 healthy volunteers of similar age and BMI (13 male, mean age 51±2.6 years; BMI 26±0.84) were studied. Patients were on oral antidiabetic therapies only, with mean HBA1c 7.4±1.3% and no history of diabetic complications. Obstructive coronary artery disease (defined as> 50% lumen diameter reduction) was excluded in all patients by CT coronary angiography. Cardiac 31P-MRS (3T) was performed at rest and during 8 minutes of leg exercise lying prone, with 2.5 kg weights attached to both legs. First-pass perfusion images (using a saturation recovery fast-gradient echo sequence and 0.03 mmol/kg bolus of Gadoterate meglumine (Dotarem, Guerbet LLC, France ) were also acquired at stress (3-6 minutes i.v. adenosine, 140 μg/kg/min) and rest at mid ventricular short axis. myocardial perfusion reserve index (MPRI) were measured from perfusion images.

## Results

Left ventricular ejection fraction (LVEF) and mass index (LVMI) were similar in patients with T2DM and healthy volunteers (LVEF 70±1 vs 71±0.9%, p=0.494; LVMI 56.7±1.8 vs 52.6±2.4g/m^2^, p=0.168). Increases in rate pressure product with exercise (T2DM 46±36%, healthy volunteers 42±26%, P = 0.69) and adenosine stress (T2DM 40 ± 3%, healthy volunteers 39 ± 3%, P = 0.89) were similar. There was no change in PCr/ATP during exercise in healthy volunteers (rest: 2.03±0.05, exercise: 2.10±0.09 P=0.84). Resting PCr/ATP was reduced in patients (1.67±0.04, P=< 0.001) compared to controls, and during exercise, there was a further reduction in PCr/ATP (1.52±0.05, P=0.037 vs rest). As expected, myocardial perfusion reserve was significantly reduced in diabetic patients (1.64±0.08 vs 2.1±0.14 in controls, P=0.002). Furthermore, there was a significant correlation between MPRI and exercise energetics (R=0.401, P=0.015), but not rest energetics.

## Conclusions

During exercise, the pre-existing energetic deficit in diabetic cardiomyopathy is further exacerbated. While myocardial energetics at rest is not related to coronary microvascular dysfunction and is primarily a result of metabolic dysfunction, during exercise, microvascular dysfunction exacerbates the energetic deficit.

## Funding

The National Institute for Health Research Oxford Biomedical Research Council and Sir Henry Dale Fellowship from the Welcome Trust and the Royal Society [Grant Number 098436/Z/12/Z] supported this work.

**Figure 1:**
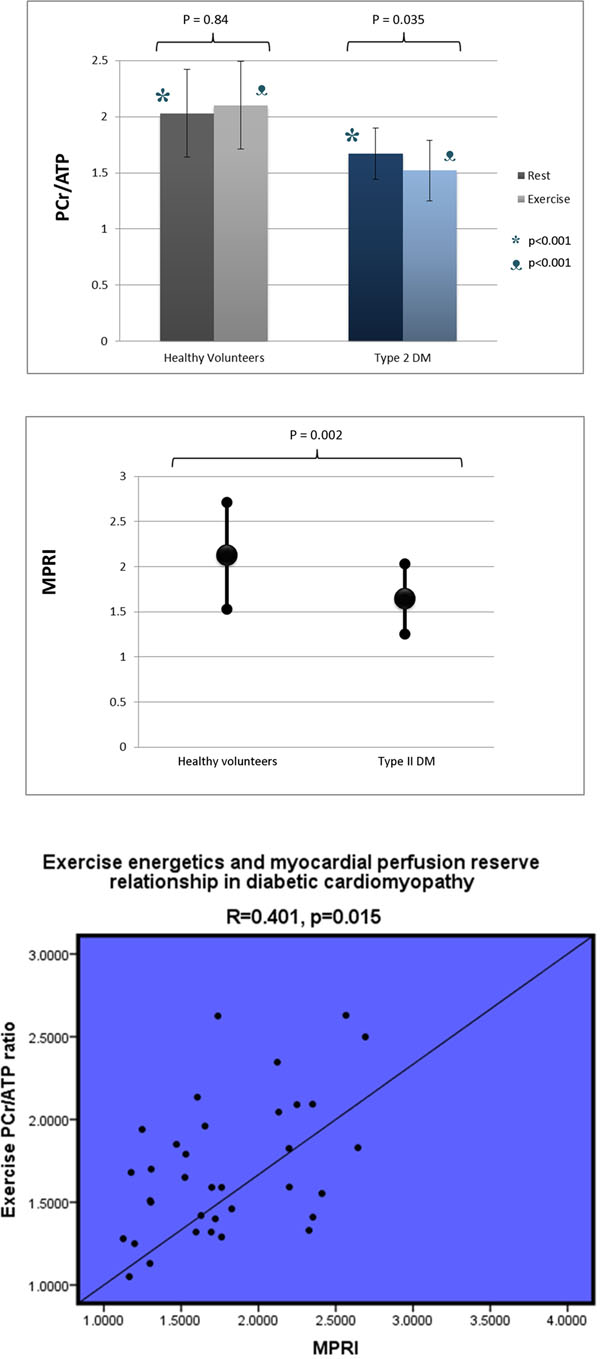
Rest and exercise myocardial energetics and myocardial perfusion reserve assessments in patients with T2DM and healthy volunteers

